# Economic Evaluation of an Intervention to Improve Mouth Care and Reduce Pneumonia Incidence in Nursing Homes: An Exploratory Analysis

**DOI:** 10.3390/nursrep16090330

**Published:** 2026-09-09

**Authors:** Sally C. Stearns, Jane A. Weintraub, John S. Preisser, Kimberly T. Ward, Christopher J. Wretman, Philip D. Sloane, Sheryl Zimmerman

**Affiliations:** 1 Gillings School of Global Public Health, The University of North Carolina at Chapel Hill, Chapel Hill, NC 27599, USA; sally_stearns@unc.edu (S.C.S.); sheryl_zimmerman@unc.edu (S.Z.); 2 The Cecil G. Sheps Center for Health Services Research, The University of North Carolina at Chapel Hill, Chapel Hill, NC 27599, USAchristopher.wretman@ed.ac.uk (C.J.W.);; 3 Adams School of Dentistry, The University of North Carolina at Chapel Hill, Chapel Hill, NC 27599, USA; 4 Duke Clinical Research Institute, Duke University, Durham, NC 27710, USA; 5 School of Social and Political Science, The University of Edinburgh, Edinburgh EH89JU, UK; 6 School of Medicine, The University of North Carolina at Chapel Hill, Chapel Hill, NC 27599, USA; 7 School of Social Work, The University of North Carolina at Chapel Hill, Chapel Hill, NC 27599, USA

**Keywords:** aged, nursing homes, economic evaluation, oral health, oral hygiene, pneumonia, hospitalizations, “Mouth Care Without a Battle”

## Abstract

**Background/Objectives**: Poor oral health, which is often overlooked in nursing homes (NHs), is associated with aspiration pneumonia. This analysis assesses the cost effectiveness of an oral hygiene intervention, “Mouth Care Without a Battle©” (MCWB), which trains NH staff to provide daily mouth care. The first year of a pragmatic cluster randomized effectiveness trial conducted in 14 NHs demonstrated a significant reduction in pneumonia incidence in intervention NHs. **Methods**: Because the two-year primary outcome was not statistically significant, the first-year analysis is post hoc. The incremental cost-effectiveness ratio (ICER) of costs (staff training, time spent providing mouth care, and pneumonia-related hospitalizations) to effects (reductions in pneumonia cases) over one year was estimated using bootstrapping. **Results**: Analyses for 1605 residents with clustering by NH show that the likelihood that MCWB is cost-saving ranges from 6.5% to 61.1% depending on whether staff are paid benefits and various levels of estimated hospital costs. Similarly, the likelihood that MCWB results in reduced pneumonia cases but with higher costs ranges from 32.0% to 86.6%. Whether MCWB is cost-effective depends on society’s willingness to pay for reduced pneumonia cases and related unmeasured benefits. These estimates may represent a lower bound of potential cost savings because analyses did not include costs of treating pneumonia if not hospitalized, ambulance transport, or the emotional costs of illness. However, other excluded costs (e.g., dental restorations) and resources required to improve fidelity could reduce the likelihood of cost savings. **Conclusions**: MCWB and similar interventions that improve oral health care in NHs may yield cost savings or produce benefits from the reduction in pneumonia that justify net increases in costs incurred. It is also important to consider who bears the costs (e.g., the NH) versus who reaps the benefits (e.g., healthier residents and reduced Medicare payments).

## 1. Introduction

Poor oral health is prevalent among older adults residing in nursing homes (NHs) and is frequently overlooked, resulting in neglected mouth care [1,2,3]. Many NH residents are independently unable to maintain adequate oral hygiene due to overall poor health and physical or cognitive impairments. Additionally, access to routine professional oral health care is often limited in these settings [4]. Consequently, responsibility for daily mouth care usually falls to NH staff.

Wang and colleagues’ 2026 literature review of oral health care delivery in NHs identified barriers for implementing good oral health practices due to various factors: workforce (e.g., inadequate education and preparation of caregivers), organizational (e.g., low priority given to oral health and lack of integration with other protocols), and policy (e.g., lack of dental insurance coverage) [5]. Providing oral care to older adults, particularly those with dementia, can be challenging. Residents with cognitive impairment may exhibit resistive behaviors when staff attempt to provide mouth care, at least in part because staff are not trained to do so [6]. More pointedly, NH staff often face competing clinical demands, inadequate training or knowledge, negative attitudes, and fear related to providing mouth care [3,7]. These challenges are consequential given that 42% of NH residents in the United States have Alzheimer’s disease or related dementias or other cognitive impairment [8,9].

Poor oral health and periodontal diseases have been associated with many adverse systemic diseases, including bacterial pneumonia [4,10]. Nursing home-acquired pneumonia (NHAP), including aspiration pneumonia, is a serious and potentially life-threatening condition that can require hospitalization. NHAP has been linked to oral bacterial pathogens colonizing dental plaque [11], which may be aspirated into the respiratory tract [12,13]. A one-year longitudinal study in France [14] using insurance data found that health care costs increased by 60–93% for NH residents who experienced at least one episode of NHAP.

Although interventions to improve oral health care in NHs require investment of time and resources, they may also reduce total costs or improve valued health outcomes. Several economic evaluations support this possibility. A cost-effectiveness analysis in Australian residential care facilities compared various scenarios of professional oral health care with current practice and found the professional care scenarios to be cost-effective, measured as cost per pneumonia case averted [15]. Similarly, a German study comparing professional oral health care with no professional care demonstrated cost effectiveness for preventing NHAP from a public–private payer perspective [16].

Several systematic reviews have examined the association between oral health and pneumonia, although not all have been limited to or included NH residents. A 2006 review found fair evidence of an association of pneumonia with oral health and good evidence that improved oral hygiene and professional oral care reduced respiratory diseases, including in NHs [17]. Likewise, a 2008 systematic review that included clinical studies and randomized clinical trials reported that oral hygiene interventions provided to older adults in hospitals and NHs reduced pneumonia incidence and may reduce mortality [18,19]. Finally, a Cochrane Database systematic review [20] assessed six randomized controlled trials evaluating oral care measures, including brushing, swabbing, denture cleaning, mouth rinse, and combinations of these, among residents in NHs and other long-term care settings. Although these studies compared professional care to usual care, they were deemed low quality and at high risk of bias. Consequently, the authors were unable to determine if professional oral care reduced the incidence of NHAP.

“Mouth Care Without a Battle©” (MCWB) is a comprehensive evidence-based program designed to educate and train NH staff to provide daily mouth care to residents [21]. The program incorporates strategies to address behaviors of residents who may be resistant or combative and to help staff who are reluctant or unsure how to provide care. An NIH-funded two-year pragmatic cluster randomized trial conducted in 14 North Carolina NHs assigned to seven matched pairs demonstrated significant improvements in oral hygiene and denture cleanliness in intervention vs. usual care sites (all *p* < 0.05) [22]. This intervention significantly reduced pneumonia incidence in the first year of the trial (incident rate ratio, 0.69; upper bound of 1-sided 95% CI, 0.94; *p* = 0.03) [23]. However, results were not significant in the second year, likely due to reduced intervention fidelity and sustainability, as suggested by daily mouth care logs and related factors [24]. In Wang and colleagues’ review of workforce intervention studies [5], a one-hour training had less effect on staff self-efficacy [25] than multifaceted approaches, such as MCWB and the Mouth Care Matters (MCM) program [26]. In a randomized clinical trial involving NH residents with dementia who resisted mouth care, an intervention was successful in facilitating mouth care but showed only small improvement in oral health [6].

This study evaluates the cost effectiveness of the MCWB intervention using data from the first year of the NIH-funded trial due to sustainability challenges in the second year. Favorable economic findings would provide additional justification for prioritizing effective mouth care interventions for NH residents who require support for daily mouth care.

## 2. Materials and Methods

Key aspects of the trial are summarized below, with full details available elsewhere [23]. Fourteen North Carolina NHs that had comparatively higher hospitalization rates for pneumonia were recruited and matched in pairs based on size and the 6-month pneumonia incidence rate. Within each pair, one NH was randomized to MCWB, in which staff were trained and supported to provide daily mouth care for two years. MCWB was developed particularly for residents with cognitive and physical impairment, although the intention was that all residents received daily mouth care. Training included in-service sessions delivered by a specialist in dementia care and dental hygiene at study onset, along with monthly support visits over two years. A booster in-service training was conducted at 12 months. All nursing assistants, nurses, and administrative staff were invited to participate. Nursing assistants and administrative staff received three hours of training, while licensed practical nurses (LPNs) and registered nurses (RNs) received an additional fourth hour focused on the broader MCWB program, including project goals, quality improvement monitoring, and staff responsibilities. In each NH, a nursing assistant was identified as a dedicated oral care aide who trained and supported new staff and cared for residents who required the most time. The salary for the dedicated aide was provided by the research grant. The aide’s main activities involved participating in training (initial session and beyond) and providing oral health care directly to residents. Residents in control NHs received standard mouth care.

The economic evaluation was planned in the original protocol, which envisioned the second year of the trial to be a “sustainability” analysis. The restriction to the first year of data for the economic evaluation was made once the second-year fidelity to the intervention was found to be sub-optimal. Thus, while the restriction of the economic evaluation to the first year of the trial was not explicitly in the original protocol, trial registration, or statistical analysis plan, the sustainability concerns meant the first year of the trial was most relevant for the economic implications. The analysis of the first year data is therefore exploratory and should be considered to be a post hoc analysis until efforts to sustain the intervention can be proven to be effective.

Medical records of all residents in and admitted to the 14 NHs during the study period were abstracted quarterly. A waiver of consent to abstract medical records was granted by the Office of Human Research Ethics of the University of North Carolina at Chapel Hill, which also approved all study procedures (IRB #13-2072). The study was registered on ClinicalTrials.gov (NCT03817450). Data collection was conducted from September 2014 to May 2017 by research assistants blinded to study group assignment.

The primary outcome was incidence of pneumonia per 1000 resident-days, with statistical power based on pneumonia incidence over two years of follow-up. Pneumonia was considered present based on a diagnosis of pneumonia of any type recorded in nurse or provider notes [23]. Distinguishing between aspiration pneumonia and other types of pneumonia was beyond the scope of the study. The study pre-specified 1-sided tests for intervention effectiveness with respect to the primary outcome due to assumption that MCWB would not increase pneumonia. Secondary resident outcomes included incidence of all-cause hospitalization, hospitalization due to pneumonia (any type), and mortality. Pneumonia-related hospitalizations were identified based on medical record charting. Pneumonia not needing hospitalization and hospitalization for pneumonia were treated as separate events. Residents could have multiple occurrences during the year.

Overall, the study enrolled 2152 residents. Among residents with non-missing data, the mean age was 79.4 years; 66.2% were women and 62.2% were white. Participants included 1219 residents (56.6%) in seven intervention NHs and 933 residents (43.4%) in seven control NHs. During the 2-year study period, the incidence rate of pneumonia per 1000 resident-days was 0.67 and 0.72 in the intervention and control NHs, respectively. Neither unadjusted nor covariate-adjusted intent-to-treat analyses found a significant reduction in pneumonia due to MCWB during the total 2 years of follow-up, but adjusted post hoc analyses limited to 1605 first year residents found a significant reduction in pneumonia incidence in intervention NHs (Incidence Rate Ratio (IRR), 0.69; upper bound of 1-sided 95% CI = 0.94; *p*  = 0.03) [23]. A total of 1684 residents were in the 14 NHs during the first year of the MCWB intervention, but 79 residents were not included in the analysis due to missing data on characteristics used in the models predicting pneumonia cases and hospitalizations. Only 4.95% of intervention NH residents and 4.34% of control NH residents were missing covariate data. Although the missingness mechanism could not be formally established, complete-case analysis was selected because the proportion of missing data was small and similar between study groups. No adjustments or imputations were made for the 79 cases with missing data.

For the economic evaluation, an incremental cost-effectiveness ratio (ICER) was estimated to determine the costs per pneumonia case avoided by MCWB NHs compared to control NHs from a social (combined public–private) perspective. While clinical studies are usually powered for health outcomes, researchers are paying increasing attention to standards for economic evaluation alongside clinical trials [27]. The Consolidated Health Economic Evaluation Reporting Standards (CHEERS) checklist [28] was used for this study and is available in the Supplementary Material (File S2).

Results of economic assessments in trials are subject to uncertainty from sampling, parameter estimates and other sources, such as imputation of missing data [27]. In economic evaluations alongside cluster RCTs, researchers should account for skewed costs and/or effects and clustering of data [29]. Analyses of trial data for individual participants should also consider both sampling uncertainty and parameter uncertainty and adjust for imbalance in baseline covariates [30].

We used a non-parametric bootstrap approach with sampling of residents for parameter estimation; the bootstrap approach accounts for correlations between the benefits/effects and costs of the intervention [31,32]. Individuals within a cluster are likely to be similar in characteristics or care received, so outcomes and costs are likely more homogeneous within rather than between clusters. Ignoring clustering in analysis likely leads to underestimation of statistical uncertainty, resulting in incorrect inference, and may lead to bias. Clustering, which was addressed in the analyses of pneumonia cases and hospitalizations for pneumonia, should also be accounted for in economic evaluation.

This analysis used bootstrapping with clustering by NH [29,30] to estimate the incremental cost-effectiveness ratio (ICER). The ICER schematically defined in Equation (1) gives the cost per pneumonia case avoided from using the MCWB approach by using the difference in costs between treatment (with MCWB) and control (without MCWB) in the numerator and the difference in effects (pneumonia cases) between treatment and control in the denominator.

(1) $$ICER=\left({Cost}_{treat}-{Cost}_{control}\right)/\left({Effect}_{treat}-{Effect}_{control}\right)\;ICER=\frac{{Cost\;\left(\begin{array}{c} oral\;care\;plus\;MCWB\;training\\ plus\;pneu\;hospitalizations \end{array}\right)}_{treat}-{Cost\;\left(\begin{array}{c} oral\;care\;plus\\ pneu\;hospitalizations \end{array}\right)}_{control}}{{Predicted\;Pneumonia\;Cases}_{control}-{Predicted\;Pneumonia\;Cases}_{treat}}$$

More detailed formulas for the estimation of the separate components of the ICER are provided in Section 3 (Results). We treated hospitalizations for pneumonia as a cost in the ICER because any reductions in hospital costs stemming from MCWB will offset the oral care costs and training costs. Because the outcome of interest is an undesirable outcome (pneumonia cases), we reversed the order of the outcomes to “control minus treatment” in the denominator in order to calculate the cost per pneumonia case avoided. Analyses were conducted using Stata Version 19.5.

Predicted values of pneumonia cases and hospitalizations due to pneumonia were obtained from the intent-to-treat regression equations estimated in the main study with covariates to adjust for random imbalance in resident covariates between treatment groups [23]. The covariates included resident age at baseline, use of eating support, whether the resident had asthma or chronic obstructive pulmonary disease, use of a feeding tube, receipt of antibiotic medication, flu vaccine documented that year, and pneumococcal vaccine up to date. The predictions were based on residents’ observed treatment assignment, and not on standardized counterfactual predictions under both the intervention and control conditions. Predicted rates were used to control for individual level covariates by adjustment for random imbalance in covariates between intervention and control arms. The original study compared unadjusted and covariate-adjusted pneumonia incidence rates side-by-side and found few differences [23], strongly suggesting that use of observed versus predicted pneumonia outcomes in the present analysis would have little impact on the results. Sampling residents while holding the original prediction model fixed may underestimate total uncertainty. However, these exploratory analyses de-emphasize statistical inference that is based on variance estimation or other quantifications of uncertainty.

Appendix A provides information on the categories of costs related to oral health care in both intervention and control NHs. Estimates of oral care costs, including training, used median hourly wage estimates for certified nursing assistants (CNAs), licensed practical nurses (LPNs), registered nurses (RNs) and the dental hygienist (i.e., the trainer) in 2019 ($14.26, $22.83, $35.24, and $36.65, respectively) [33,34]. The analysis assumed a staff salary benefit rate of 30% (for health insurance and retirement) so as not to understate costs, even though NH staff do not always receive benefits at this rate. Therefore, we also provide an estimate of the ICER assuming that staff did not receive benefits. Appendix B provides information on the oral care supply costs for intervention and control NHs. The Medicare cost for hospitalization for pneumonia was estimated from the Healthcare Cost and Utilization Project (HCUPnet) [35]. Because the estimated Medicare cost of a hospital stay for pneumonia varies substantially across the nation and depends on whether the patient has complications or comorbidities (CCs) or major complications or comorbidities (MCCs), we used both a low and high estimated cost: the low value was $4943, which is the estimated cost of a Medicare hospital stay for simple pneumonia and pleurisy without CC/MCC in North Carolina in 2019; the high value of estimated Medicare costs was $11,115, the national US estimate for simple pneumonia with MCC. Appendix C provides details of the specifications used in HCUPnet. Discounting was not used because costs and effects were only measured for one year. All cost measures and estimates are in 2019 US dollars.

**Figure 1:**
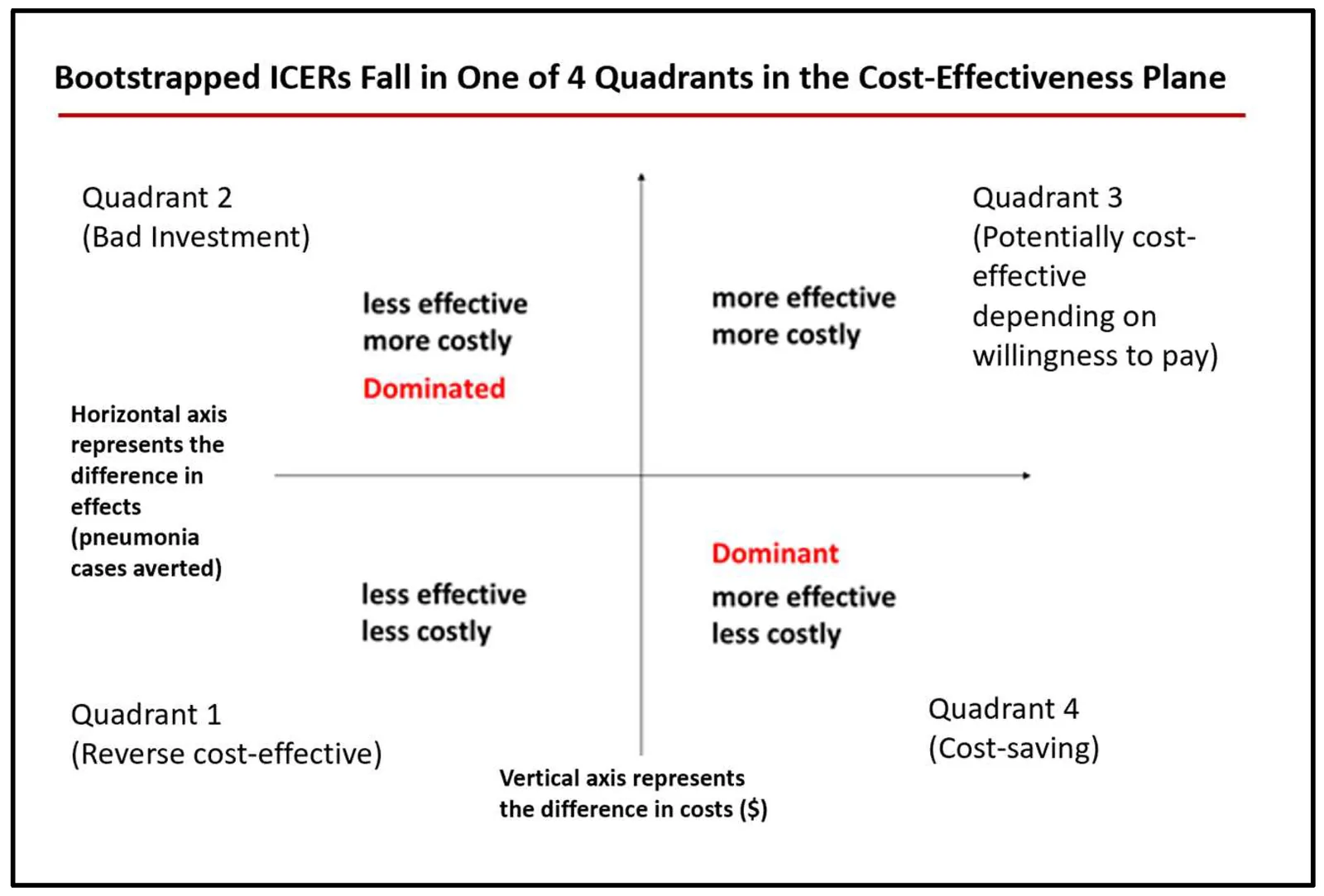
Cost-effectiveness plane with four quadrants [36].

Bootstrapping provided the distribution of ICER estimates in the cost-effectiveness plane, as shown in Figure 1. The four quadrants (Q) of the plane, as typically represented in the health economics literature [36], indicate whether the intervention is cost-saving (Q4 or southeast/dominant corner), more costly and more effective (Q3 or northeast corner, where an estimate would be considered cost effective if it falls below a Willingness-to-Pay (WTP) threshold in this quadrant), or not desirable due to an increase in pneumonia cases from the intervention (Q1 or Q2 in the southwest and northwest/dominated corners, respectively). Dominant means that costs decreased while effects improved (cost-saving), and dominated means that costs increased while effects decline (a very undesirable situation).

Bootstrapping replications to estimate the likelihood that the intervention was cost-saving (i.e., reduced pneumonia cases and reduced net costs) were obtained using 10,000 iterations. Supplementary File (File S1) contains the Stata program used to construct the analysis files and the bootstrapping algorithm used for the ICER replications. The ICER replications fall into one of the four quadrants based on whether net costs increased or decreased, and whether net effects (pneumonia cases avoided) increased or decreased. For replication estimates that fall in Quadrant 3 (more effective and greater net costs), we estimated the median cost per pneumonia case averted (i.e., 50% of the estimates in Quadrant 3 fell above a line from the origin, and 50% fell below that line). Specifying a WTP threshold for averting a case of pneumonia is beyond the scope of this analysis. However, should a decision maker have a value they are willing to pay for averting a case of pneumonia, the slope of a line with that value could be used to determine which of the estimates in Quadrant 3 are deemed to be cost effective (i.e., the reduction in pneumonia cases is worth the additional cost). The analysis provides a cost-effectiveness acceptability curve (CEAC) to the probability of the intervention being cost effective at different willingness-to-pay thresholds.

The bootstrapping used stratification by treatment status and sampled clusters (NH sites) with replacement. Generally, whether a statistical analysis should preserve the pair matching depends on the trial’s goals, sample size, and the specific statistical framework being used to handle cluster-level variance. However, bootstrapping cannot create external validity beyond that provided by the sampling frame and study design. Although adjusting for clustering has been shown to be theoretically and practically important, variance estimation in economic evaluation may not be robust in cluster randomized trials when the number of clusters is small and there are unequal numbers of observations per cluster [37]. We broke the pair matching for the seven pairs in the bootstrapping because information loss, as represented by the loss of degrees of freedom from pair matching, can become a particularly critical consideration in small cluster randomized trials involving 10 pairs or less [38]. The analysis choice in favor of more stable estimation with bootstrapping clusters by breaking pairs involved a trade-off. As cluster size (using the proxy of NH bed count) was a stratification factor in the matched-pair design, the resampling of clusters separately within intervention and control strata relinquished balancing on cluster size in the analysis. While the choice to break pair matching in the bootstrapping may have increased variability, our analysis de-emphasized variance estimation by focusing on other parameters (quadrants) of the ICER distribution.

## 3. Results

The first year of the intervention included 1605 resident observations, with complete data on resident characteristics, the number of days observed, and actual and predicted outcome information. Rates of pneumonia cases per 1000 resident-days were 0.66 and 0.91 in the intervention and control NHs, respectively; rates of hospitalizations for pneumonia per 1000 resident-days were 0.24 and 0.28 in the intervention and control NHs, respectively.

The economic evaluation results are presented in two parts: (1) a detailed description of the calculation of ICER, and (2) ICER estimation results with and without staff benefits and for low and high estimates of the cost of a hospital stay for pneumonia.

### 3.1. Constructing the ICER

The components of ICER presented in Equation (1) (above) were implemented using more detailed formulas, listed below, for components of the ICER numerator (Equations (2) and (3)) and the ICER denominator (Equation (4)).

(2) $${Cost}_{treat}=\left(\frac{\sum_{j=1}^{N_{t}}\sum_{i=1}^{n_{{treat}_{j}}}\left({days}_{ij}*\left({pr(oc)}_{treat}\left({oc\$}_{treat}+{train\$}_{{treat}_{j}}\right)\right){+pred\_hosp}_{ij}*hosp\$\right)}{\sum_{j=1}^{N_{t}}\sum_{i=1}^{n_{{treat}_{j}}}\left({days}_{ij}\right)}\right)$$

(3) $${Cost}_{control}=\left(\frac{\sum_{j=1}^{N_{c}}\sum_{i=1}^{n_{{cont}_{j}}}\left({{{days}_{ij}*pr\left(oc\right)}_{cont}*oc\$}_{cont}{+pred\_hosp}_{ij}*hosp\$\right)}{\sum_{j=1}^{N_{c}}\sum_{i=1}^{n_{{cont}_{j}}}{days}_{ij}}\right)$$

(4) $${Effect}_{control}-{Effect}_{treat}=\left(\frac{\sum_{j=1}^{N_{c}}\sum_{i=1}^{n_{cont}}{pneu\_cases}_{ij}}{\sum_{j=1}^{N_{c}}\sum_{i=1}^{n_{cont}}{days}_{ij}}\right)-\left(\frac{\sum_{j=1}^{N_{t}}\sum_{i=1}^{n_{treat}}{pneu\_cases)}_{ij}}{\sum_{j=1}^{N_{t}}\sum_{i=1}^{n_{treat}}{days}_{ij}}\right)$$

In the ICER equation components (Equations (2)–(4) above), j indexes NH sites, i indexes residents within sites, $N_{t}$ is the number of treatment sites, $N_{c}$ is the number of control sites, $n_{{treat}_{j}}$ is the number of residents sampled in the j-th treatment site, and $n_{{cont}_{j}}$ is the number of residents sampled in the j-th control site. The costs (Equations (2) and (3)) and the difference in effects (Equation (4)) are per resident-day. Table 1 provides descriptive statistics and values, as well as definitions for various components within these formulas using the variable names in Equations (2)–(4).

The first four rows in Table 1 are fixed values estimated from the study data that do not vary by resident. The first two rows are average values for treatment and control NHs based on estimates of the amount of time spent providing mouth care for both treatment and control NHs. The study did not record the receipt or length of mouth care for each resident throughout the study. These estimates are based on observations conducted by the research assistant of mouth care provided by two randomly selected CNAs to approximately 12 residents over two mornings approximately six months after the initial training. The data therefore are derived from 28 CNAs at 14 nursing homes, with an average of 12 residents per NH, all over two separate days. The research assistant shadowed these two CNAs, documenting time spent per resident and completeness of mouth care based on the MCWB protocol. Most of the difference in the cost per mouth care session (line 2 in Table 1) was due to the longer time aides spent providing mouth care in intervention versus control NHs (see Appendix A and Appendix B). To avoid an intervention effect from this activity, the research assistant recorded time for all personal care tasks provided during morning care (e.g., dressing) so as not to suggest that mouth care was of special interest.

The MCWB training costs (third line in Table 1) were estimated separately for each treatment nursing home based on data on staff participation in training sessions. Overall, 47% of nursing aides and 42% of RN/LPN staff attended either the three- or four-hour training plus refresher trainings, respectively (data available on request). The training costs per mouth care session were calculated using estimated total mouth care sessions, which also depended on the number of residents in an NH (i.e., larger NHs had lower training costs per session).

The low and high estimates of cost for hospitalization for pneumonia (fourth line in Table 1) were described earlier in Section 2 and Appendix C. The costs of hospital stays were assumed not to vary based on NH treatment status.

The last three rows in Table 1 were measured or estimated at the resident level and therefore vary by resident. Resident-days were measured for each resident as part of the original data collection. The predicted values of the number of pneumonia cases and the number of hospitalizations for pneumonia came from the negative binomial regressions estimated for these outcomes in the main study. These models adjusted for the seven resident-level covariates indicated earlier, with descriptive statistics available in the main study [23]. These predicted values were used to calculate and bootstrap the ICER.

### 3.2. ICER Estimation with Treatment Stratification and Clustering by NH

Table 2 shows results for the main bootstrapped ICER estimations with stratification by treatment status (MCWB), with and without staff receiving benefits, such as health insurance and retirement (valued at 30% of wages).

**Figure 2:**
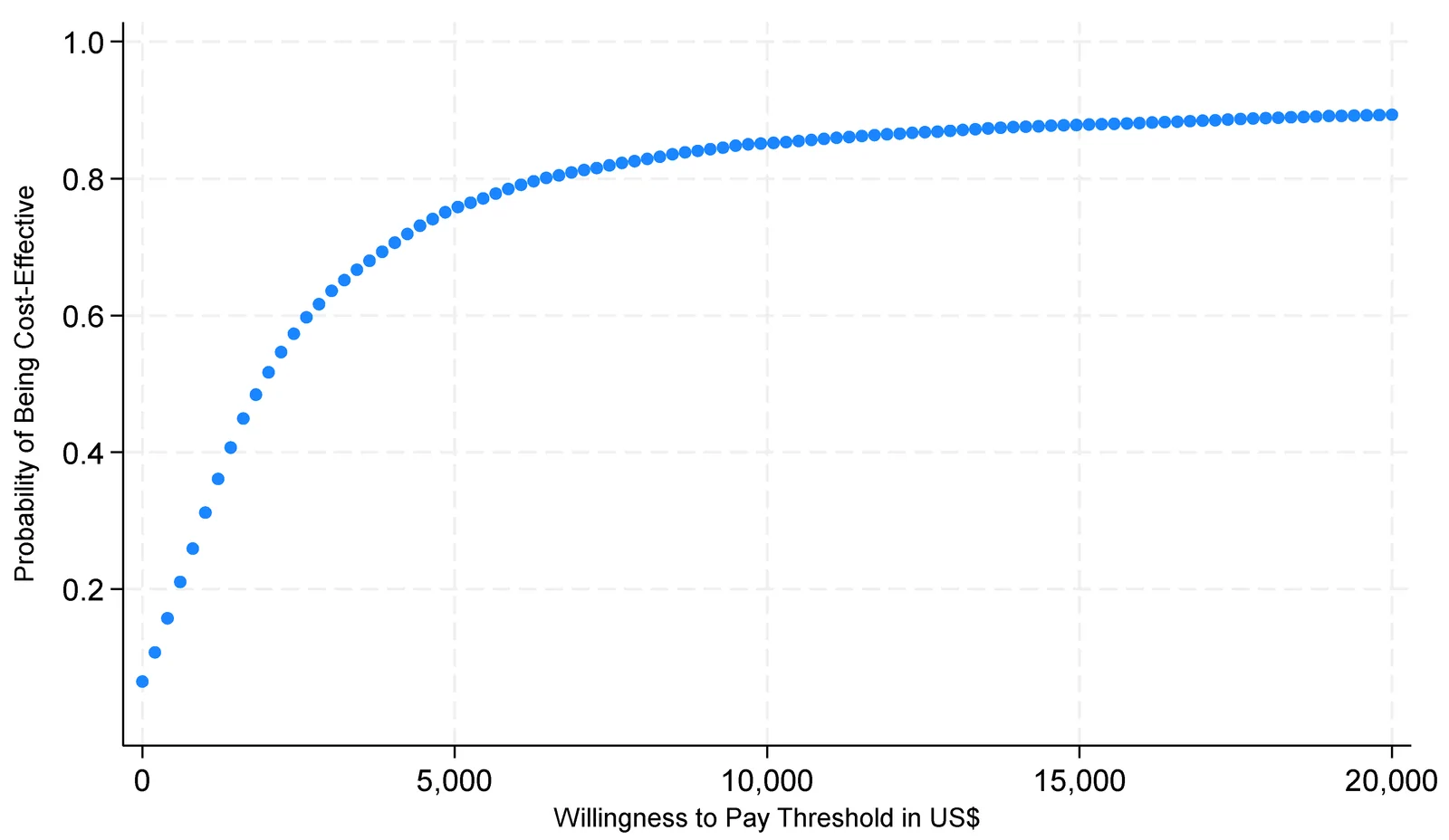
CEAC assuming employee benefits equal to 30% of wages and hospital stay cost of $4943.

The results that use a staff benefit rate of 30% and the low estimated cost of a hospital stay ($4943) show a 6.5% likelihood that the intervention is cost-saving (Q4, or dominant in the sense that costs decreased and effects improved) and a 86.6% likelihood that the intervention is more effective but more costly (Q3, where effects are improved but costs are higher) when examined in relation to the pneumonia and hospitalization outcomes used in these analyses. The probability that the intervention is cost effective in Q3 depends on the WTP threshold per pneumonia case avoided. The analysis indicates a small (6.9%) likelihood that the intervention results in both higher costs and more pneumonia cases, and 0.0% chance that lower costs but more pneumonia cases result. The CEAC in Figure 2 shows the distribution of ICER estimates for WTP ranging from $0 (i.e., the likelihood of being cost-saving) to over $20,000 before ultimately reaching the asymptote at the 93.1% level (i.e., the sum of 6.5% plus 86.6%). The asymptote reflects the proportion of bootstrap replications with a favorable incremental effect as the willingness-to-pay threshold becomes very large. The 86.6% of ICER estimations showing the intervention has higher net costs for reduced pneumonia cases (Q3) have a median cost of $1897 per pneumonia avoided by MCWB (last column). In Q3, the mean incremental cost per 1000 resident-days per year was $213, and the mean incremental benefit (pneumonia cases avoided) was 0.105 per 1000 resident-days per year. However, as shown by the CEAC in Figure 2, a large proportion of the ICER estimates in Q3 have quite high values; the 95th percentile incremental cost per pneumonia case avoided for Q3 is more than $17,000, and the unconditional mean estimate for the ICER estimates falling in Q3 is $14,968.

The second row shows the results under the assumption that staff do not receive paid benefits in addition to their wage salary while still assuming the low hospitalization cost. Accordingly, MCWB is more likely to be cost-saving (16.2%) and is 76.9% likely to have higher costs but better effects with a median incremental cost per pneumonia case avoided of $1464. The CEAC for these estimates is very similar to Figure 2, except for the higher value of 16.2% for the WTP of $0 (the point where the line intersects the Y axis). However, attaining a higher likelihood of being cost-saving because staff who may already be underpaid are not receiving benefits does not seem to be socially desirable.

As shown in the third and fourth lines of Table 2, using the high hospital stay cost estimate substantially increases the likelihood of MCWB being cost-saving (50.2% when staff benefits are paid at 30%, and 61.2% when staff are not paid benefits). The likelihood of having higher costs and better effects decreases accordingly. However, in all four cases, the likelihood of being dominated because costs increase and effects are worse is slightly less than 7%. Therefore, the CEAC is very similar for all four cases, differing only by the vertical axis intercept, which represents the likelihood of being cost-saving.

## 4. Discussion

When clinical trials show evidence of effectiveness, it is important that they are accompanied by economic evaluation [23,29]. While the original clinical trial was powered based on two-year pneumonia incidence, the significant reduction in pneumonia cases during the first year combined with observations that fidelity to the intervention was compromised in the second year [24] justify economic evaluation of only the first-year trial results. The economic evaluation adjusting for clustering by NH shows that the likelihood that MCWB is cost-saving ranges from 6.5% to 61.1% (depending on whether staff are paid benefits and the estimated costs of hospital stays for pneumonia) when examined in relation to pneumonia cases and hospitalization. The likelihood that the intervention increases costs and reduces pneumonia cases ranges from 32.0% to 86.6%. Whether MCWB is cost-effective in these instances depends on willingness to pay to avoid a pneumonia case. However, some costs and benefits were not included, and data on the presence of complications and comorbidities for residents hospitalized with pneumonia would be needed to provide a more precise estimate of MCWB being cost-saving or potentially cost effective. In total, the analysis of first-year data is exploratory and should be considered to be a post hoc analysis until efforts to sustain the intervention can be proven to be effective.

Of note, several important costs that are not included are the cost of treating non-hospitalized pneumonia cases, cost of ambulance transfer, and emotional costs, such as reductions in quality of life for patients, staff and families. Assuming each of these costs is higher in control NHs than in treatment NHs, the estimates from the main analysis likely underestimate the potential cost savings from MCWB. For example, costs for treating pneumonia in the NH can vary greatly depending on severity, comorbidities, and type of treatment provided. A 2003 study of the costs of treating pneumonia in an NH estimated the cost at $458 but noted that the costs varied widely depending on clinical presentation [39]. Incorporation of this estimate in the analysis would increase the likelihood that MCWB is cost-saving. This consideration combined with hypothesized ambulance costs and value of emotional costs can be weighed informally against the median costs per pneumonia case avoided. It is, however, very hard to value the emotional costs to patients, staff and families. Furthermore, some costs were not included, such as the potential costs of dental treatment resulting from the greater scrutiny of oral health brought about by MCWB.

It is important to note that the estimated average effects over the 14 participating NHs do not reflect the expected experience for individual NHs. Variation in results by NH could be caused by factors including variation across NHs in easily observed measures, such as size or staff participation in training, fidelity to MCWB, and resident oral hygiene and morbidity/risk, among others. Variation in program effectiveness and cost effectiveness could also be caused by unobserved factors, such as the skills and effectiveness of the staff person assigned to oversee the training and implementation of MCWB, the management within the treatment NHs, or differences in the unmeasured/controlled case complexity of residents across NHs, such as underlying medical conditions or socio-economic status. That said, it is logical to surmise that providing training to staff in a larger NH has the potential to benefit more residents given economies of scale, and that staff can further train and support each other, a model that MCWB promotes. Formal investigation of heterogeneity requires an appropriate multilevel or cluster-level framework and must be interpreted cautiously given the small number of nursing homes.

Due to methodologic limitations, the results are best considered exploratory and illustrative rather than as a definitive assessment of the cost effectiveness of MCWB to improve mouth care in NHs. The short-term effectiveness of MCWB under supported implementation may not be sustainable under routine nursing home conditions, as evidenced by the problems of fidelity and sustainability during the second year of the program [24]. The observation of time spent by staff providing mouth care was limited in this study. Furthermore, it is important to restate that the study specifically recruited NHs with comparatively higher hospitalization rates for pneumonia. Therefore, the results cannot be generalized to all NHs.

While the estimation adjusted for uncertainty generated by resampling residents clustered by NH, the analysis did not account for other potentially important sources of uncertainty. Specifically, the estimation did not adjust for: uncertainty in the prediction-model coefficients; uncertainty in fixed cost and resource-use input estimates; structural uncertainty arising from the selected analytical assumptions. Therefore, the reported probabilities are conditional on the fixed model coefficients and cost assumptions.

Use of multiple imputation and sensitivity analyses on a number of parameters used in the study (e.g., staff wages) could be insightful in future studies. Mortality was not significantly affected by the intervention and is not incorporated in the analysis. Pneumonia was ascertained based on chart diagnosis, which could either undercount cases (e.g., due to incomplete documentation) or overcount cases (e.g., due to misinterpretation of infiltrate on chest X-ray). Data were not collected on whether each resident received oral health care or for what length of time, so estimates were constructed based on logs for residents cared for by two CNAs at the beginning of the study. Selecting the period with a statistically significant clinical result may introduce time-window selection or post hoc analysis bias. Finally, data are from 2014–2017, and costs from the time of the main outcome analysis (2019) are not inflated to the current year. Shifts in relative prices over time or pneumonia incidence outbreaks, such as during the COVID-19 pandemic, could change results over time.

The study has numerous strengths and raises some important issues. The idea of improving mouth care for NH residents is inherently appealing from a societal perspective. The majority of older adults are not covered by dental insurance, and traditional Medicare does not cover dental services except for a few hospital-based services, such as organ transplants, where removing oral infection and inflammation is deemed “medically necessary.” In many states, Medicaid dental benefits minimally cover dental services for adults or do not cover them at all. Without dental coverage, cost becomes a major deterrent to obtaining dental care. The social perspective used in this analysis is beneficial because it combines public and private costs, and it addresses prevention rather than treatment. However, the analysis does not include all public or private costs or benefits. Also, the analysis does not distinguish between who bears the cost of the intervention (e.g., costs to the NH for extra staff training in a non-trial setting) versus who reaps the benefit (e.g., reduced Medicare payments from reduced hospitalizations, improved oral health and reduced dental care and other health-related expenses, not included in this analysis). Without some attention to this issue, NHs might bear the cost of implementing a program but would not benefit from reductions in Medicare hospitalization payments.

Furthermore, obtaining data for oral health cost analyses can be challenging because administrative claims data are not available. Data from longitudinal studies, such as MCWB, that document oral conditions and episodes of pneumonia and hospitalizations are noteworthy. For NH residents, oral health assessments reported in the Minimum Data Set (MDS) are usually completed by non-dentally trained staff, lack standardization, do not report oral hygiene status, and under-report needed dental care [40]. The MCWB study benefited from having an experienced research dental hygienist collect clinical oral health data and a trained research team abstract pneumonia and hospitalization information from residents’ NH charts.

In total, the findings are indicative of a high likelihood that MCWB may result in either cost-saving or benefits for which society may be willing to pay—especially considering that the analysis focused only on the cost and benefits of residents avoiding pneumonia and did not assess other concomitant oral health benefits of clean teeth, including reduction in dental caries, periodontal diseases, tooth loss, associated systemic diseases and conditions affected by infection and inflammation in the mouth, and improved quality of life. However, some costs were not included (e.g., dental restorations), and the resources required to improve fidelity could increase costs and reduce the likelihood of cost savings. Theoretically, dental treatment costs should be incurred with or without the intervention, but disease might not be identified and treated until further advanced, resulting in either more complex and expensive treatment or extraction and tooth loss. Therefore, the results do not imply that the intervention definitively demonstrated cost savings because the estimates originate from a post hoc analysis and do not use a lifetime perspective or include all costs or benefits.

The analysis is also instructive for future trials and economic evaluations of mouth care interventions. If MCWB is cost saving or cost effective in NHs (depending on willingness to pay), hospitals might also consider using it to hire or co-locate dental hygienists to teach staff to provide daily mouth care to patients who require support. If the results seen in NHs are similar, implementation of the program in hospital settings could potentially reduce non-ventilator hospital-acquired pneumonia. Supporting this point, the U.S. Veterans Health Administration has implemented a similar program, the Hospital-Acquired Pneumonia Prevention by Engaging Nurses (HAPPEN) initiative [41], reflecting that oral hygiene and oral health are important in any setting. While the economic evaluation of oral health interventions in other settings would require separate study, this exploratory analysis of MCWB could provide a framework for such an evaluation. Additionally, research on willingness to pay for avoiding pneumonia cases could be a useful direction, especially for countries that make program funding decisions based on economic evaluation.

## Figures and Tables

**Table 1:** Cost parameters and descriptive data from MCWB intervention for ICER estimation.

Parameter Name	Description	Treatment Value, Range or Mean (SD) N = 922	Control Value, Range or Mean (SD) N = 683
pr(oc)	Probability that a resident received daily mouth care per day (see Appendix A)	0.39	0.16
oc$	Cost per mouth care session based on minutes per session and certified nursing assistant wage rate plus supplies (see Appendix A and Appendix B)	$2.41	$1.14
train$	Estimated MCWB training costs for each estimated total mouth care session	NH range: $0.32 to $0.67	0
hosp$	Estimated Medicare cost per pneumonia hospitalization (see Appendix C)	Low ($44,943) High ($11,115)	Low ($44,943) High ($11,115)
days	Resident-specific number of days in NH	230 (140)	242 (123)
pred_hosp	Resident-level predicted number of pneumonia hospitalizations (range: 0–2)	0.058 (0.065)	0.063 (0.056)
pneu_cases	Resident-level predicted number of pneumonia cases per participant (range: 0–4)	0.181 (0.172)	0.195 (0.166)

Note: SD = standard deviation (in parentheses for mean values). $ represents US dollars.

**Table 2:** Bootstrapped ICER distribution (10,000 iterations) with resampling of NH clusters.

Quadrant	4	3	2	1	
	Cost Saving	Potentially Cost Effective *	Bad Investment	Reverse CE **	Median Cost Per Pneumonia Case Avoided if Quadrant 3
Hospital Stay Cost: $4943 Staff Benefits 30%	6.5%	86.6%	6.9%	0.0%	$1897
No Staff Benefits	16.2%	76.9%	6.9%	0.0%	$1464
Hospital Stay Cost: $11,115 Staff Benefits 30%	50.2%	43.0%	6.8%	0.2%	$2143
No Staff Benefits	61.1%	32.0%	6.4%	0.5%	$1966

Note: All estimates are based on data for 1605 residents. The four quadrants (Q1–Q4) are defined in Figure 1 and were determined based on whether incremental costs increased or decreased, and whether incremental benefits increased or decreased. Generally, people view increases in incremental benefits (Q3 or Q4) as favorable and decreases in incremental benefits (Q1 or Q2) as unfavorable. * Estimates in Quadrant 3 represent estimates with higher cost and better effect; therefore, whether an estimate is cost-effective is based on the WTP estimate specified by a social decision-maker. ** Reverse CE means costs were lower but effects were worse (more pneumonia cases).

## Data Availability

The datasets presented in this article are not readily available because of the confidentiality of NH resident-level protected health information (PHI). Requests to access the datasets should be directed to Sheryl Zimmerman at the University of North Carolina at Chapel Hill.

## Supplementary Materials

The following supporting information can be downloaded at: https://www.mdpi.com/article/10.3390/nursrep16090330/s1. File S1: Stata Program Code; File S2: CHEERS Checklist.

## Public Involvement Statement

We thank the residents, families, and staff who participated in this project and in the Collaborative Studies of Long-Term Care for their commitment to improving care and outcomes in nursing homes. NH staff, residents, and their families were not involved in the study design, analysis or dissemination of the study.

## Guidelines and Standards Statement

This manuscript was drafted against the Consolidated Health Economic Evaluation Reporting Standards (CHEERS) checklist.

## Abbreviations

The following abbreviations are used in this manuscript:

CC/MCC	Complication or comorbidity/major complication or comorbidity
CEAC	Cost-effectiveness acceptability curve
CNA	Certified nursing assistant
MCWB	Mouth Care Without a Battle
ICER	Incremental cost-effectiveness ratio
LPN	Licensed practical nurse
NH	Nursing home
NHAP	Nursing home-acquired pneumonia
Q1-Q4	Quadrants 1–4 in the cost-effectiveness plane
Pneu	Pneumonia
RN	Registered nurse
WTP	Willingness-to-Pay

## Appendix A. Cost Components for MCWB Training and Oral Care

The chart below shows components of the cost of the MCWB training and oral care, including wage rates and sources.

**Table A1:** Components of the MCWB training costs and oral care costs.

Item	Method for Calculation
Staff member training per staff member (intervention NHs only)	Assumes 1–3 h per staff person; one hour for initial training session, and 2 h hands-on training/refresher. Model uses cost per resident per year. 47% of CNAs and 42% of RN/LPN attended training
Cost of dental hygienist and project oral health aide for conducting training (intervention NHs only)	Assumes 9 h per intervention NH for dental hygienist and for oral health aide hired for the project (1 h prep, 1–3 h for basic staff training session, 4 h for supervisor sessions (RN/LPN) and 3 h follow-up refresher training). An additional 48 h for monthly 4 h visits by the dental hygienist were also included.
Fixed cost of MCWB training materials (intervention NHs only)	Used $75 fee for obtaining MCWB training video, available from https://www.mouthcarewithoutabattle.org/, accessed on 1 September 2026
Probability of getting oral care each day (intervention and control NHs)	Intervention NHs: 0.39 Source: Used post-intervention daily checklist data on 86,335 cases to determine the probability of getting oral care each day. Control NHs: 0.16 Source: Rate from literature: Coleman & Watson, 2006. [42]
Minutes per oral care session (intervention and control NHs)	Intervention NHs: 6.7 min Control NHs: 3.5 min Used data from MCWB pilot study, which found “Performance time increased by an average of 3.2 min, from 3.5 min at baseline to 6.7 min after the intervention (*p* < 0.001].” [21]
Cost of oral health supplies (intervention and control NHs)	Intervention NHs: $0.34 Control NHs: $0.06 See Appendix B for calculations
Hourly wage rate for Certified Nursing Assistant (CNA) This rate was also used for the project-supplied oral health aide	Bureau of Labor Statistics: U.S. Department of Labor, Nursing home certified nursing assistants, May 2019. Nursing Assistants (https://www.bls.gov/oes/2019/may/oes311131.htm, accessed on 1 September 2026) Hourly median wage was $14.26
Hourly wage rate for Licensed Practical Nurse (LPN)	Bureau of Labor Statistics, U.S. Department of Labor, Occupational Outlook Handbook, Licensed Practical and Licensed Vocational Nurses, May 2019. Licensed Practical and Licensed Vocational Nurses (https://www.bls.gov/oes/2019/may/oes292061.htm, accessed on 1 September 2026) Hourly median wage was $22.83
Hourly wage rate for Registered Nurse (RN)	Bureau of Labor Statistics: U.S. Department of Labor, Registered Nurses, May 2019. Registered Nurses (https://www.bls.gov/oes/2019/may/oes291141.htm, accessed on 1 September 2026) Hourly median was $35.24
Hourly wage rate for dental hygienist	Bureau of Labor Statistics: U.S. Department of Labor, Dental Hygienists, May 2019. Dental Hygienists (https://www.bls.gov/oes/2019/may/oes291292.htm, accessed on 1 September 2026) Hourly median was $36.65
Cost per mouth care session based on minutes per session and certified nursing assistant wage rate plus supplies (oc$ in Table 1)	Multiply CNA hourly rate times benefits (1.3) times the minutes spent providing care (minutes per hour) plus supply costs from Appendix B. Intervention(with benefits): $2.41=14.26∗1.3∗(6.7/60) + 0.34 Control (with benefits): $1.14=14.26∗1.3∗(3.5/60) + 0.06

## Appendix B. Oral Care Supply Costs

The chart below shows the oral care supply costs calculated for the study. More detailed information is available from the senior author.

**Table A2:** Oral supply costs.

Product	Cost per Item			Intervention–Daily Cost per Resident per Day		
Toothbrush	$1.08	Control cost per resident day		Interdental brush	$0.01	$0.00
Chlorhexidine 16oz	$7.55	toothpaste	$0.05	Cotton-tip applicator	0.01	0.0043
Biotene 16oz	$7.98	toothbrush	$0.01	Sponge	0.14	0.14
Listerine 3.2oz	$1.24	Total cost	$0.06	Tray covers	0.04	0.04
Interdental Brush	$0.98			Medicine cups	0.01	0.007996
Fluoride Paste: Rx (clinpro 1oz)	$3.75			Toothbrush	0.01	0.01
Fluoride Paste: Non-Rx (ACT mouthwash 16oz)	$4.99			Chlorhexidine 16oz	$0.14	$0.10
Cotton-tip applicator	$0.01			Biotene 16oz	$0.15	$0.01
Sponge	$0.14			Listerine 3.2oz	$0.11	$0.01
Tray covers	$0.04			Fluoride Paste–Rx (clinpro 4oz)	$0.05	$0.02
Medicine cups	$0.01			Fluoride Paste–Non-Rx (ACT mouthwash 16oz)	$0.09	$0.00
Toothpaste (senosodyne) 0.8oz	$1.44			Toothpaste	0.05	0.00345
Assuming 0.8oz lasts 30 days (1 brush per day)				**Total cost per person per day**		$0.34

## Appendix C. Hospital Cost Stay Estimates from HCUPnet

This appendix provides the specifications of the query of the Healthcare Cost and Utilization Project (HCUPnet), available at HCUPnet Data Tools–Healthcare Cost and Utilization Project (HCUPnet). The query, which was run on 27 August 2026, provides estimated Medicare costs per stay and Medicare charges per stay for simple pneumonia with and without complications or comorbidities. We obtained estimates for 2019 in both North Carolina and the United States. As expected, Medicare hospital charges are substantially more than Medicare costs. Because it is not known whether hospitalized patients had complications or comorbidities, we used a low value of estimated Medicare costs ($4943, the North Carolina value for simple pneumonia and pleurisy without CC/MCC) and a high value of estimated Medicare costs ($11,115, the national US estimate for simple pneumonia with major complications or comorbidities). Selected values are highlighted in yellow.

**Table A3:** Results of HCUPnet query for 2019 Medicare hospital costs and charges, 2019.

State	Principal Diagnosis	Outcome	Measure Value
North Carolina	193: Simple pneumonia and pleurisy with MCC	Average Hospital Charges per Stay (in $)	$29,085
North Carolina	193: Simple pneumonia and pleurisy with MCC	Average Hospital Costs per Stay (in $)	$8407
North Carolina	194: Simple pneumonia and pleurisy with CC	Average Hospital Charges per Stay (in $)	$21,152
North Carolina	194: Simple pneumonia and pleurisy with CC	Average Hospital Costs per Stay (in $)	$6273
North Carolina	195: Simple pneumonia and pleurisy without CC/MCC	Average Hospital Charges per Stay (in $)	$16,812
North Carolina	195: Simple pneumonia and pleurisy without CC/MCC	Average Hospital Costs per Stay (in $)	$4943
United States	193: Simple pneumonia and pleurisy with MCC	Average Hospital Charges per Stay (in $)	$45,165
United States	193: Simple pneumonia and pleurisy with MCC	Average Hospital Costs per Stay (in $)	$11,115
United States	194: Simple pneumonia and pleurisy with CC	Average Hospital Charges per Stay (in $)	$29,874
United States	194: Simple pneumonia and pleurisy with CC	Average Hospital Costs per Stay (in $)	$8183
United States	195: Simple pneumonia and pleurisy without CC/MCC	Average Hospital Charges per Stay (in $)	$22,385
United States	195: Simple pneumonia and pleurisy without CC/MCC	Average Hospital Costs per Stay (in $)	$6748
