# Supplementary material for: Economic Evaluation of an Intervention to Improve Mouth Care and Reduce Pneumonia Incidence in Nursing Homes: An Exploratory Analysis
Source: Nurs Rep. 2026 Sep 9;16(9):330. doi: 10.3390/nursrep16090330 (PMC13610211; doi:10.3390/nursrep16090330)
Supplement: Supplementary file 1 [file nursrep-16-00330-s001.zip › File S1 Stata Program Code.pdf]

File S1: Stata Program Code

```
*Mouthcare Cost-Effectiveness Analysis Stata Program

*Last updated 8/28/2026

*Contact Sally_Stearns@unc.edu if any questions


*Set the program to the directory with study files

cd "C:\Users\sstearns\OneDrive - University of North Carolina at Chapel Hill\Documents\HHS Laptop 2021\HPM\Mouthcare\Nursing Reports 2026\Nursing Reports Revision 1\stata_reanalysis"


*Selected lines must be changed below to set whether the analysis will use staff benefits at 30%

*and whether a high versus low cost of pneumonia hospital stay will be used.


*log using bs_with_benefits_r2_lowhcost, replace

log using bs_no_benefits_r2_lowhcost, replace


set more off


*First, read in the staff training data and calculate total staff training costs per site

import excel "MC_Staff_Training_scs.xlsx", firstrow clear

drop st_rnlpn*

drop if id_site==.

sum


sort id_site

list id_site st_cna*
```

```
list id_site st_lpn*

list id_site st_rn*

save training, replace


*Now get main file with predicted outcomes (pneumonia cases and pneumonia hospitalizations)

import excel "HS022298 Year 1 Resident-Level Pneumonia Hospitalization & Pneumonia.xlsx", firstrow clear

sum

gen missing=hosp_pneu_1_pred==.

tab mc missing, col row


*Calculate the rates of pneu and hosp for pneu by MC for the full first year sample

preserve

collapse (sum) days_1 hosp_pneu_1 pneu_1, by(mc)

list

gen pneu_per_1kdays=pneu_1*1000/days_1

gen hosp_per_1kdays=hosp_pneu_1*1000/days_1

list

restore


*Now keep the obs for the complete case analysis

keep if hosp_pneu_1_pred ~=.

sum

sort mc

by mc: sum
```

```
*Note that people can have more than two hospitalizations or pneumonia cases.
tab hosp_pneu_1 pneu_1, missing

*Calculate the rates of pneu and hosp for pneu by MC for complete case analysis
preserve
collapse (sum) days_1 hosp_pneu_1 pneu_1, by(mc)
list
gen pneu_per_1kdays=pneu_1*1000/days_1
gen hosp_per_1kdays=hosp_pneu_1*1000/days_1
list
restore


*Oral care variables
gen pr_oral=0.39 if mc==1
replace pr_oral=.16 if mc==0
gen min_oral=6.7 if mc==1
replace min_oral=3.5 if mc==0
gen supplies=0.34 if mc==1
replace supplies=0.06 if mc==0

*pneumonia hospitalization costs for different value of hospital costs
gen cost_hosp=4943
*gen cost_hosp=11115
gen pred_hosp_cost=hosp_pneu_1_pred*cost_hosp
```

*Now merge the files to calculate oral staff training costs per session of oral care actually provided

*(for now, calculate training cost per oral care session provided using days)

sort id_site

by id_site: egen tot_site_days=sum(days_1)

sum tot_site_days

merge m:1 id_site using training

tab _merge

*Create staff cost and training cost variables using median wages in 2019

gen wage_cna=14.26

gen wage_lpn=22.83

gen wage_rn=35.24

gen wage_denhyg=36.65

*Activate one of the two lines below to control for whether staff receive benefits at a rate of 30%

*gen benefit_rate=1.3

gen benefit_rate=1

gen hc_cna=wage_cna*benefit_rate

gen hc_lpn=wage_lpn*benefit_rate

gen hc_rn=wage_rn*benefit_rate

gen hc_denhyg=wage_denhyg*benefit_rate

*The following line uses data on NH staffing to create the MCWB training cost at each intervention NH

```
*Dental hygienist and aide training lead hours and $75 were added 8/3/2026

*Additional dental hygienist hours added 8/12/2026 for monthly NH visits of 4 hours

gen train_cost= ///
   (st_cna_1s+2*st_cna_2s+3*st_cna_3s)*hc_cna ///
        +(st_lpn_1s+2*st_lpn_2s+3*st_lpn_3s+4*st_lpn_4s)*hc_lpn ///
        +(st_rn_1s+2*st_rn_2s+3*st_rn_3s+4*st_rn_4s)*hc_rn ///
        +9*hc_cna+9*hc_denhyg ///
        +75+48*hc_denhyg


*Training costs are zero at all control NH
        replace train_cost=0 if mc==0


*Key line creating training cost per oral care session (note:  does not vary by NH)
gen train_per_oc=train_cost/(pr_oral*tot_site_days)
tab train_per_oc, missing
sort id_site
by id_site: sum train_per_oc



*calculate oral cost per session (does not include training cost)
gen oc_cost=(min_oral/60)*hc_cna+supplies


preserve
sum
bys id_site:  keep if _n==1
list mc id_site train_per_oc train_cost tot_site_days pr_oral oc_cost
```

```
restore

*Estimate the cost per pneumonia case avoided during the first year

*For denominator, weight predicted cases by the number of days
gen pneu_pcw=pneu_1_pred

*For numerator, first determine oral care costs provided ply oral care costs by number of days provided
*Then weight costs the number of days and also multiply oral care costs by number of days provided
gen costs_pcw=days_1*(pr_oral*(oc_cost+train_per_oc))+pred_hosp_cost

gen iter=1
replace iter=1

*Generate nursing home pairing for NH pair analysis
gen nh_pair=0
replace nh_pair=1 if id_site==10 | id_site==21
replace nh_pair=2 if id_site==12 | id_site==11
replace nh_pair=3 if id_site==15 | id_site==14
replace nh_pair=4 if id_site==16 | id_site==18
replace nh_pair=5 if id_site==19 | id_site==17
replace nh_pair=6 if id_site==25 | id_site==22
replace nh_pair=7 if id_site==23 | id_site==24

tab nh_pair
```

save finalfile, replace

*Now define the ICER program

program drop _all

program define my_icer

*The following line of code creates two observations, one for treatment and one for control

*It is giving mean pneumonia cases and mean costs

collapse iter cost_hosp benefit_rate oc_cost pneu_pcw costs_pcw (sum) tot_days=days_1, by(mc)

list

*ICER Calculation: By multiplying by negative 1 differently by treatment status, we are simply implementing the fact that the subtration reverses in the denominator since pneu cases is an undesirable outcome

*ICER numerator calculation

replace costs_pcw=costs_pcw*(-1) if mc==0

gen numer_cost=costs_pcw/tot_days

*ICER Denominator calculation

replace pneu_pcw=pneu_pcw*(-1) if mc==1

gen denom_cases=pneu_pcw/tot_days

list

```
gen rats=1


collapse iter cost_hosp benefit_rate (sum) dif_cost_num=numer_cost
dif_pneu_denom=denom_cases , by(rats)

gen cost_ppcase_avoided=dif_cost_num/(dif_pneu_denom)

gen quadrant=0

replace quadrant=1 if dif_cost_num<0 & dif_pneu_denom<0

replace quadrant=2 if dif_cost_num>=0 & dif_pneu_denom<0

replace quadrant=3 if dif_cost_num>=0 & dif_pneu_denom>0

replace quadrant=4 if dif_cost_num<0 & dif_pneu_denom>0


*Check descriptive stats and specific values

sum

list

list cost_hosp benefit_rate cost_ppcase_avoided quadrant dif_*



end



*First get the main estimate

preserve


my_icer

save icer_results, replace
```

```
restore
sum iter
save tempdata, replace

*Code for bootstrapping the standard errors follows
*local iter=1
while iter<10000 {

use tempdata, clear
quietly replace iter=iter+1
quietly save tempdata, replace
*Use the line below for the site paired analysis which is no longer in the manuscript
*bsample, strata(id_site)
*Use the line below for the main analysis
bsample, strata(mc) cluster(id_site)
quietly my_icer
quietly append using icer_results
*sum
quietly save icer_results, replace

}

sum cost_hosp benefit_rate quadrant
tab quadrant
sum cost_ppcase_avoided if quadrant==3, detail
```

```
*Generate the CEAC
*Graph uses a maximum WTP of $25,000
range Threshold_values 0 20000 100
g double acc = .
forval i = 1/100 {
   qui g t`i' = Threshold_values[`i'] * dif_pneu_denom > dif_cost_num
   qui sum t`i', meanonly
   replace acc = r(mean) in `i'
   drop t`i'
}


*save bs_with_benefits_r2_lowhcost, replace
save bs_no_benefits_r2_lowhcost, replace

*The lines below facilitate graph reformatting if any cosmetic changes are needed.
clear
use bs_with_benefits_r2_lowhcost
*use bs_no_benefits_r2_lowhcost

format Threshold_values %9.0fc

twoway (scatter acc Threshold_values), yscale(range(0 1)) saving(bs_benefits, replace) ///
   ylab(0(5000)20000) xtitle("Willingness to Pay Threshold in US$", size(3)) ///
         ylab(.2(.2)1, format(%4.1f)) ytitle("Probability of Being Cost-Effective", size(3))
```

```
*title(" " "Figure 2: CEAC Assuming Employee Benefits Equal to 30% of Wages and Hospital Stay Cost of $11,115", position(6) ring(1) size(3))


*twoway (scatter acc Threshold_values), yscale(range(0 1)) saving(bs_no_benefits, replace) ///
   ylab(0(5000)20000) xtitle("Willingness to Pay Threshold in US$", size(3)) ///
        ylab(.2(.2)1) ytitle("Probability of Being Cost-Effective", size(3)) ///
        title(" " "Figure 3: CEAC Assuming No Employee Benefits Paid and Hospital Stay Cost of $4,943", position(6) ring(1) size(3))


*Get mean incremental cost and mean incremental effect per year per 1000 residents
gen inc_cost_py_p1kres=dif_cost_num*365*1000
gen inc_eff_py_p1kres=dif_pneu_denom*365*1000
sum inc_cost_py_p1kres inc_eff_py_p1kres if quadrant==3, detail



log close
```
