# Supplementary material for: Economic Evaluation of an Intervention to Improve Mouth Care and Reduce Pneumonia Incidence in Nursing Homes: An Exploratory Analysis
Source: Nurs Rep. 2026 Sep 9;16(9):330. doi: 10.3390/nursrep16090330 (PMC13610211; doi:10.3390/nursrep16090330)
Supplement: Supplementary file 1 [file nursrep-16-00330-s001.zip › File S2 CHEERS Checklist.pdf]

ISPOR
Improving healthcare decisions

Consolidated Health Economic Evaluation Reporting Standards (CHEERS) 2022 Checklist

The CHEERS 2022 statement replaces the 2013 CHEERS statement, which should no longer be used. The CHEERS 2022 checklist contains 28 items with accompanying descriptions. Checklist users should indicate the section of the manuscript where relevant information can be found. The authors recommend using a section heading with a paragraph number. If an item does not apply to a particular economic evaluation, checklist users are encouraged to report "Not Applicable." If information is otherwise not reported, checklist users are encouraged to write, "Not Reported." Users should avoid the term "Not Conducted" as CHEERS is intended to guide and capture reporting. Additional information on CHEERS 2022 can be found here.

Title

1. Title

Identify the study as an economic evaluation and specify the interventions being compared.

First page of article: Economic Evaluation of an Intervention to Improve Mouth Care and Reduce Pneumonia Incidence in Nursing Homes: An Exploratory Analysis (The intervention is Mouth Care Without a Battle, and the comparison is to usual oral care in nursing homes.)

Abstract

2. Abstract

Provide a structured summary that highlights context, key methods, results, and alternative analyses.

First page of article: Background/Objectives: Poor oral health, which is often overlooked in nursing homes (NHs), is associated with aspiration pneumonia. This analysis assesses the cost-effectiveness of an oral hygiene intervention, "Mouth Care Without a Battle©" (MCWB) that trains NH staff to provide daily mouth care. The first year of a pragmatic cluster randomized effectiveness trial conducted in 14 NHs demonstrated a significant reduction in pneumonia incidence in intervention NHs.
Methods: Because the two-year primary outcome was not statistically significant, the first-year analysis is post hoc. The incremental cost-effectiveness ratio (ICER) of costs (staff training, time spent providing mouth care, and pneumonia-related hospitaliza-tions) to effects (reductions in pneumonia cases) over one year was estimated using bootstrapping.

© 2022 ISPOR — The Professional Society for Health Economics and Outcomes Research
International Society for Pharmacoeconomics and Outcomes Research, Inc

Results: Analyses for 1,605 residents with clustering by NH show the likelihood that MCWB is cost-saving ranges from 6.5% to 61.1% depending on whether staff are paid benefits and various levels of estimated hospital costs. Similarly, the likelihood that MCWB results in reduced pneumonia cases but with higher costs ranges from 32.0% to 86.6%. Whether MCWB is cost-effective depends on society's willingness to pay for reduced pneumonia cases and related unmeasured benefits. These estimates may rep-resent a lower bound on potential cost savings because analyses did not include costs of treating pneumonia if not hospitalized, ambulance transport, and emotional costs of illness. However, other excluded costs (e.g., dental restorations) and resources required to improve fidelity could reduce the likelihood of cost savings.
Conclusions: MCWB and similar interventions that improve oral health care in NHs may yield cost savings or produce benefits from the reduction in pneumonia that justify net increases in costs incurred. It is also important to consider who bears the costs (e.g., the NH) versus reaps the benefits (e.g., healthier residents and reduced Medicare payments).

Introduction

3. Introduction: Background and Objectives

Give the context for the study, the study question, and its practical relevance for decision making in policy or practice.

Section: 1. Introduction (Paragraph 6): "Mouth Care Without a Battle©" (MCWB) is a comprehensive evidence-based program designed to educate and train NH staff to provide daily mouth care to residents. This study evaluates the cost-effectiveness of the MCWB intervention using data from the first year of the NIH-funded trial due to sustainability challenges in the second year. Favorable economic findings would provide additional justification for prioritizing effective mouth care interventions for nursing home (NH) residents who require support for daily mouth care.

Methods

4. Health economic analysis plan

Indicate whether a health economic analysis plan was developed and where available.

Section: 2. Materials and Methods (Paragraph 2): The economic evaluation was planned in the original protocol, which envisioned the second year of the trial to be a "sustainability" analysis. The restriction to the first year of data for the economic evaluation was made once the second year fidelity to the intervention was found to be sub-optimal. Thus, while the restriction of the economic evaluation to the first year of the trial was not explicitly in the original protocol, trial registration, or statistical analysis plan, the sustainability concerns meant the first

year of the trial was most relevant for the economic implications. The analysis of the first year data is therefore exploratory and should be considered to be a post hoc analysis until efforts to sustain the intervention can be proven to be effective.

5. Study population

Describe characteristics of the study population (such as age range, demographics, socioeconomic, or clinical characteristics).

Section: 1. Introduction (Paragraph 6) and 2. Materials and Methods (Paragraph 5): An NIH-funded two-year pragmatic cluster randomized trial conducted in 14 North Carolina NHs assigned to seven matched pairs demonstrated significant improvements in oral hygiene and denture cleanliness in intervention vs. usual care sites. Overall, the study enrolled 2,152 residents; among residents with non-missing data, the mean age was 79.4 years, 66.2% were women and 62.2% were white. Partic-ipants included 1219 residents (56.6%) in seven intervention NHs and 933 residents (43.4%) in seven control NHs. During the 2-year study period, the incidence rate of pneumonia per 1000 resident-days was 0.67 and 0.72 in the intervention and control NHs, respectively.

6. Setting and location

Provide relevant contextual information that may influence findings.

Section: 2. Materials and Methods (Paragraph 1): Fourteen North Carolina NHs that had comparatively higher hospitalization rates for pneumonia were recruited and matched in pairs based on size and 6-month pneumonia incidence rate.

7. Comparators

Describe the interventions or strategies being compared and why chosen.

Section: 2. Materials and Methods (Paragraph 1): Within each pair, one NH was randomized to MCWB in which staff were trained and supported to provide daily mouth care for two years. MCWB was developed particularly for residents with cognitive and physical impairment, although the intention was that all residents receive daily mouth care.

8. Perspective

State the perspective(s) adopted by the study and why chosen.

Section: 2. Materials and Methods (Paragraph 6): The study considered both private and public (Medicare) costs. The intent was to use a societal perspective, though some benefits and costs were not included because of lack of availability of data. The study identifies the type of costs and benefits that are not included.

---

9. Time horizon

State the time horizon for the study and why appropriate.

Section: 2. Materials and Methods (Paragraph 3): Study recruitment of nursing homes occurred in 2014. The enrollment and follow-up of residents in the 14 nursing homes occurred from 2014-2017.

---

10. Discount rate

Report the discount rate(s) and reason chosen.

Section: 2. Materials and Methods (Paragraph 11): Not applicable. Discounting was not used because costs and effects were only measured for one year.

---

11. Selection of outcomes

Describe what outcomes were used as the measure(s) of benefit(s) and harm(s).

Section: 2. Materials and Methods (Paragraph 4): The main outcome was the occurrence of cases of pneumonia in NH residents during the two year follow-up period in each NH. Secondary resident outcomes included incidence of all-cause hospitalization, hospitalization due to pneumonia (any type), and mortality.

---

12. Measurement of outcomes

Describe how outcomes used to capture benefit(s) and harm(s) were measured.

Section: 2. Materials and Methods (Paragraph 3): Medical records of all residents in and admitted to the 14 NHs during the study period were abstracted quarterly.

---

13. Valuation of outcomes

Describe the population and methods used to measure and value outcomes.

© 2022 ISPOR — The Professional Society for Health Economics and Outcomes Research
International Society for Pharmacoeconomics and Outcomes Research, Inc

Section: 2. Materials and Methods (Paragraph 6): The outcome of "pneumonia cases avoided" was not valued. Therefore, certain aspects of the outcome, such as emotional benefits of not having pneumonia for the residents, their families, and facility staff, were not captured in the analysis. Measures such as quality-adjusted life years were not relevant both because of the one year follow-up time frame for the study as well as the lack of use of an instrument measuring the value of avoiding a case of pneumonia in the original study.

---

14. Measurement and valuation of resources and costs

Describe how costs were valued.

Section: 2. Materials and Methods (Paragraph 9): Costs included training costs for the intervention, costs of oral care (treatment and control homes, including cost of oral care supplies), and costs of hospitalization for pneumonia. Estimates of oral care costs including training used median hourly wage estimates for certified nursing assistants (CNAs), licensed practical nurses (LPNs), registered nurses (RNs) and the dental hygienist (i.e., the trainer) in 2019 ($14.26, $22.83, $35.24, and $36.65 respectively). The analysis assumed a staff salary benefit rate of 30% (for health insurance and retirement) so as not to understate costs even though NH staff do not always receive benefits at this rate. NHs. The Medicare cost for hospitalization for pneumonia was estimated from the Healthcare Cost and Utilization Project (HCUPnet) [35]. Because the estimated Medicare cost of a hospital stay for pneumonia varies substantially across the nation and by whether the patient has complications or comorbidities (CC) or major complications or comorbidities (MCC), we used both a low and high estimated cost: the low value was $4,943, which is the estimated cost of a Medicare hospital stay for simple pneumonia and pleurisy without CC/MCC in North Carolina in 2019, and the high value of estimated Medicare costs ($11,115, the national US estimate for simple pneumonia with MCC.

---

15. Currency, price date, and conversion

Report the dates of the estimated resource quantities and unit costs, plus the currency and year of conversion.

Section: 2. Materials and Methods (Paragraph 11): All cost measures or estimates are in 2019 US dollars.

---

16. Rationale and description of model

If modeling is used, describe in detail and why used. Report if the model is publicly available and where it can be accessed.

Section: 3. Results (Paragraph 6): We did not use modeling (e.g., such as Markov modeling) because of the one year time frame for follow-up. We used parametric negative binomial regression analysis to model getting

pneumonia and having a hospitalization for pneumonia as a function of resident characteristics: resident age at baseline, use of eating support, whether the resident had asthma or chronic obstructive pulmonary disease, use of a feeding tube, receipt of antibiotic medication, flu vaccine documented that year, and pneumococcal vaccine up to date. Predicted values for these two outcomes were used in bootstrapping estimation of the incremental cost-effectiveness ratio (ICER).

---

17. Analytics and assumptions

Describe any methods for analyzing or statistically transforming data, any extrapolation methods, and approaches for validating any model used.

Section: 2. Materials and Methods (Paragraph 9): We used a non-parametric bootstrap approach with sampling of residents for parameter estimation; the bootstrap approach accounts for correlations between the benefits/effects and costs of the intervention. Individuals within a cluster are likely to be similar in characteristics or care received, so outcomes and costs are likely more homogeneous within rather than between clusters. Ignoring clustering in analysis likely leads to underestimation of statistical uncertainty, resulting in incorrect inference, and may lead to bias. Clustering, which was addressed in the analyses of pneumonia cases and hospitalizations for pneumonia, should also be accounted for in economic evaluation. Also, see the published original outcome study: Zimmerman, S.; Sloane, P.D.; Ward, K.; Wretman, C.J.; Stearns, S.C.; Poole, P.; et al. Effectiveness of a mouth care program pro-vided by nursing home staff vs standard care on reducing pneumonia incidence: A cluster randomized trial. JAMA Netw. Open. 2020, 3, e204321–e204321. 10.1001/jamanetworkopen.2020.4321

---

18. Characterizing heterogeneity

Describe any methods used for estimating how the results of the study vary for subgroups.

Not applicable, as subgroup analysis was not conducted.

---

19. Characterizing distributional effects

Describe how impacts are distributed across different individuals or adjustments made to reflect priority populations.

Section: I. Introduction (Paragraph 2): The focus of the study was on a priority population: aged NH residents who are known to often receive sub-optimal oral care.

---

20. Characterizing uncertainty

Describe methods to characterize any sources of uncertainty in the analysis.

Section: 2. Materials and Methods (Paragraph 8): We used a non-parametric bootstrap approach with sampling of residents for pa-rameterestimation. The bootstrap approach accounts for correlations between the benefits/effects and costs of the intervention. The original study compared unadjusted and covariate-adjusted pneumonia incidence rates side-by-side and found few differences, strongly suggesting that use of ob-served versus predicted pneumonia outcomes in the present analysis would have little impact on the results. Sampling residents while holding the original prediction model fixed may underestimate total uncertainty. However, these exploratory analyses deemphasize statistical inference that is based on variance estimation or other quantifications of uncertainty.

---

21. Approach to engagement with patients and others affected by the study

Describe any approaches to engage patients or service recipients, the general public, communities, or stakeholders (eg, clinicians or payers) in the design of the study.

Not applicable: Residents and their families were not engaged in the design of the study.

Results

22. Study parameters

Report all analytic inputs (eg, values, ranges, references) including uncertainty or distributional assumptions.

Section: 2. Materials and Methods (Paragraph 10): Predicted values of pneumonia cases and hospitalizations due to pneumonia were obtained from the intent-to-treat regression equations estimated in the main study with covariates to adjust for random imbalance in resident covariates between treatment groups. Key parameters such as measures of the probability a resident received oral care and the time spent on oral care were estimated separately for treatment versus control NHs but did not vary within treatment group due to lack of detailed data. Training costs were estimated separately for each intervention NH because of differences in NH size. Data on complications and comorbidities for residents who were hospitalized for pneumonia were not available, so Medicare costs for pneumonia stays were estimated using HCUPnet; a low and high value of hospital cost was used to assess the range of ICER values.

---

23. Summary of main results

Report the mean values for the main categories of costs and outcomes of interest and summarize them in the most appropriate overall measure.

© 2022 ISPOR — The Professional Society for Health Economics and Outcomes Research

International Society for Pharmacoeconomics and Outcomes Research, Inc

Section: 3. Results (Section 3.2, Paragraph 2): The results that use a staff benefit rate of 30% and the low estimated cost of a hospital stay ($4,943) show a 6.5% likelihood that the intervention is cost-saving (or dominant in the sense that costs decreased and effects improved) and a 86.6% likeli-hood that the intervention is more effective but more costly when examined in relation to the pneumonia and hospi-talization outcomes used in these analyses. The probability that the intervention is cost-effective in depends on the WTP threshold per pneumonia case avoided. The analysis indicates a small (6.9%) likelihood that the intervention results in both higher costs and more pneumonia cases, and 0.0% chance that lower costs but more pneumo-nia cases result. A Cost-Effectiveness Acceptibility Curve (not shown here) shows the distribution of ICER estimates for WTP ranging from $0 (i.e., the likelihood of being cost-saving) to over $20,000 before ultimately reaching the asymptote at the 93.1% level (i.e., the sum of 6.5% plus 86.6%). The asymptote reflects the proportion of bootstrap replications with a favorable in-cremental effect as the willingness-to-pay threshold becomes very large. The 86.6% of ICER estimations showing the intervention has higher net costs for reduced pneumonia cases have a median cost of $1,897 per pneumonia avoided by MCWB (last col-umn). As shown by the CEAC, a large proportion of the ICER estimates have quite high values; the 95th percentile incremental cost per pneumonia case avoided for Q3 is more than $17,000, and the unconditional mean es-timate for the ICER estimates falling for ICER estimates where costs increased but pneumonia cases were reduced is $14,968.

---

24. Effect of uncertainty

Describe how uncertainty about analytic judgments, inputs, or projections affects findings. Report the effect of choice of discount rate and time horizon, if applicable.

Section: 2. Materials and Methods (Paragraphs 10-14): The use of bootstrapping the ICER was the main approach to addressing uncertainty. As noted earlier, the use of many fixed parameters makes this analysis exploratory rather than definitive.

---

25. Effect of engagement with patients and others affected by the study

Report on any difference patient/service recipient, general public, community, or stakeholder involvement made to the approach or findings of the study.

Not applicable.

Discussion

26. Study findings, limitations, generalizability, and current knowledge

Report key findings, limitations, ethical, or equity considerations not captured and how these could impact patients, policy, or practice.

Section: 4. Results (Paragraphs 1-10; the following text is from the concluding 10th paragraph): The analysis is also instructive for future trials and economic evaluations of mouth care interventions. If MCWB is cost-saving or cost-effective in NHs (depending on willingness to pay), hospitals might also consider using it to hire or co-locate dental hygienists to teach staff to provide daily mouth care to patients who require support. If the results seen in NHs are similar, implementation of the program in hospital settings could potentially reduce non-ventilator hospital-acquired pneumonia. Supporting that point, the U.S. Veterans Health Administration has implemented a similar program, the Hospital-Acquired Pneumonia Prevention by Engaging Nurses (HAPPEN) initiative reflecting that oral hygiene and oral health are important in any setting. While the economic evaluation of oral health interventions in other settings would require separate study, this exploratory analysis of MCWB could provide a framework for such an evaluation. Additionally, research on willingness to pay for avoiding pneumonia cases could be a useful direction, especially for countries that make program funding decisions based on economic evaluation.

Other Relevant Information

27. Source of funding

Describe how the study was funded and any role of the funder in the identification, design, conduct, and reporting of the analysis.

Additional information at the end of the manuscript: This research was supported by grant R01HS022298 from the Agency for Healthcare Research and Quality (AHRQ).

---

28. Conflicts of interest

Report authors' conflicts of interest according to journal or International Committee of Medical Journal Editors requirements.

Additional information at the end of the manuscript: Drs. Zimmerman and Sloane are university-based researchers, which assists in dissemination of Mouth Care Without a Battle. The Mouth Care Without a Battle website provides a video for training at $75 (inclusive of administration and postage). There are no licensing implications nor marketing expenses, and the economic evaluation was conducted independently with AHRQ funding noted above. Dr. Weintraub, Dr. Stearns, Dr Zimmerman and Dr. Sloane reported receiving grants from the National Institutes of Health during the conduct of the study. No other disclosures were reported.

© 2022 ISPOR — The Professional Society for Health Economics and Outcomes Research

International Society for Pharmacoeconomics and Outcomes Research, Inc
